# Ultraviolet radiation and mitochondrial bioenergetic effects by reactive oxygen species production

**DOI:** 10.4103/mgr.MEDGASRES-D-25-00171

**Published:** 2026-03-17

**Authors:** Aline S. Perez, Natalia M. Inada, Vanderlei S. Bagnato

**Affiliations:** Institute of Physics, University of São Paulo, São Paulo, Brazil; São Carlos Institute of Physics, University of São Paulo, São Carlos, Brazil; Biomedical Engineering Department, Texas A&M University, College Station, TX, USA

Mitochondria are widely recognized as the primary cell energy generators, producing adenosine triphosphate (ATP) via oxidative phosphorylation. However, their role extends far beyond energy metabolism. These organelles are central hubs in regulating redox balance, calcium signaling, cell cycle progression, and the intrinsic pathway of apoptosis. They are complex structures of varying size and shape, on average 1 μm long, and possess their own DNA (mitochondrial DNA) originating exclusively from the maternal genetic content.^1^ These organelles consume approximately 90% of cellular oxygen and produce more than 80% of the energy needed to maintain the metabolic life of cells. They have two lipid membranes: the outer membrane, with a composition similar to the cell’s, and the inner membrane, which forms invaginations called cristae to increase its surface area. The protein complexes of the electron transport chain and ATP synthase are concentrated within these cristae. **Figure 1** illustrates some mitochondrial events induced by ultraviolet (UV) radiation that affect the electron transport chain, such as swelling and mitochondrial proton leak. In recent years, increasing research has reframed mitochondria not just as metabolic engines but as active participants in cellular adaptation, stress responses, and immune modulation. As such, mitochondrial dysfunction has emerged as a key player in the pathogenesis of a wide array of diseases, including neurodegenerative disorders such as Parkinson’s and Alzheimer’s diseases, cardiovascular conditions, metabolic syndromes, and various forms of cancer.^2^ UV radiation has been used as a treatment for certain chronic skin conditions, such as psoriasis and urticaria, due to its ability to reduce inflammation and manage disease symptoms. These clinical applications have been well documented.^3^ However, similar to X-rays, UV radiation exposure can be harmful to nuclear DNA or mitochondrial DNA integrity, and several adverse side effects have been reported, ranging from erythema to carcinogenesis.^4^ Conversely, insufficient exposure to ultraviolet B (UVB) radiation may impair the conversion of 7-dehydrocholesterol into vitamin D in the skin, a process that depends on the energy absorbed from this wavelength. Most studies involving UV radiation and mitochondria focus on its ionizing potential and its effects on genetic alterations. Others investigate the intramitochondrial production of reactive oxygen species in response to varying doses of ultraviolet A (UVA). It is also known that not only UV light, but also blue light can disrupt mitochondrial function, increase reactive oxygen species production and alter mitochondrial bioenergetics. Despite the therapeutic value of UV radiation, it is widely used in disinfection and sterilization systems for surfaces and food. Microorganism inactivation by UV is one of the most common methods employed by the food industry for preservation.^5^ The biological consequences of electromagnetic radiation, especially UV light, have long been associated with DNA damage and skin-related pathologies. However, recent evidence reveals that mitochondria dynamic regulators of cellular metabolism and death also exhibit notable sensitivity to specific UV wavelengths, such that ultraviolet C (UVC) radiation causes measurable bioenergetic dysfunction in isolated mitochondria, specifically those derived from liver tissue. This perspective aims to contextualize some findings, highlight the implications for photobiology and mitochondrial research, and suggest future investigative avenues.

**Figure 1:**
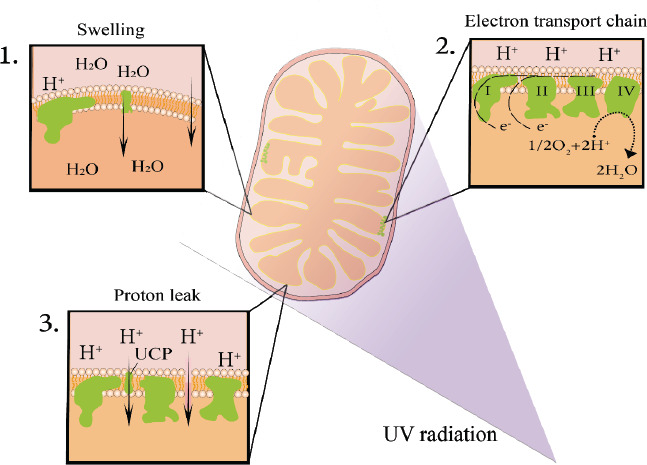
Alterations in mitochondrial components can lead to changes in normal mitochondrial activities due to UV radiation exposure. (1) Mitochondrial swelling, (2) the electron transport system, and (3) proton leak. Created with Adobe Illustrator software. UCP: Uncoupling protein; UV: ultraviolet.

**UV radiation effect in mitochondrial bioenergetics:** Given their multifaceted roles, mitochondria are also sensitive sensors and potential targets of environmental stressors including electromagnetic radiation, drugs, oxygen gas availability and temperature which can unfold proteins one of the major contents of mitochondria.^6^,^7^ Thus, different external elements can cause changes in the selective permeability of the internal membrane. Among these, UV radiation is particularly relevant due to its natural ubiquity and artificial applications, ranging from phototherapy to sterilization protocols. UV light has long been associated with DNA damage and skin carcinogenesis, yet its impact on mitochondria has received comparatively less attention. Recent advances suggest that mitochondria may be directly affected by UV exposure in ways that bypass classical nuclear pathways. This includes modulation of respiratory efficiency, induction of mitochondrial swelling (a marker of membrane permeability transition), and decoupling of ATP production. Furthermore, mitochondrial damage may be a primary mechanism underlying the antimicrobial effects of UVC radiation, especially in single-celled organisms and eukaryotic pathogens. In this context, studies that promote investigating mitochondrial bioenergetics in response to UVA and UVC radiation can be relevant in understanding the effects of this electromagnetic radiation range.

In a recent study, by our group, the high-resolution respirometry and swelling assays were carried out to investigate mitochondrial bioenergetics post-irradiation with UVA (371 nm) and UVC (255 nm) light in different doses.^8^ In the reaction chambers the amount of oxygen is fixed, with gas consumption carried out exclusively by the organelles. It is known that UVA radiation exposure does not lead to a statistically significant change in mitochondrial bioenergetic effects, immediately after irradiation, in isolated mice liver mitochondria incubated in reaction medium, in the presence of substrates in any dose. While under UVC radiation exposure and in the same experimental conditions, the mitochondria were affected not only in their bioenergetics but also demonstrated a dose-dependent response; that is, the dysfunction increased as the dose increased. Demonstrating that after irradiation, in different doses, it is observed less preservation of the coupling of mitochondrial functions and therefore less oxidative phosphorylation may occur, so fewer ATP molecules are formed. Besides that, it is already known that after UVC irradiation there are limitations of the oxidative phosphorylation system such as electron transport system and ATPase activity, another disfunction observed indicates energy losses, dissipated during electron transport, thus, there is a loss of the electron transport chain ability to effectively carry electrons, the low activity of the electron transport chain will also determine the production of ATP, since there may be imbalances in the membrane potential due to the lower pumping of protons out of the matrix.^9^

**Dose-dependent mitochondrial dysfunction due UVC exposure:** We observed that the bioenergetic decrease under UVC is not abrupt but followed a dose-dependent pattern. Coupling efficiency, and uncoupling control ratio decreased linearly with increasing UVC exposure.^8^ This quantitative consistency strengthens the causative link between UVC photons and mitochondrial decoupling. Importantly, these effects occurred in isolated mitochondria, where nuclear DNA and cellular signaling pathways are absent. Thus, the data suggests a direct photophysical or photochemical interaction between UVC photons and mitochondrial structures possibly involving lipids, proteins in the electron transport chain, or the inner membrane potential. In contrast, UVA exposure even at doses as high as 756 mJ/cm² did not alter respiration parameters. This wavelength-selective response underscores the importance of photon energy: UVC photons, having higher energy, are more likely to induce structural damage or disrupt electron transport components, while UVA may lack sufficient energy to trigger such changes, at least in the absence of photosensitizers.

**Implications for phototherapy and sterilization:** This knowledge is particularly relevant in biomedical contexts involving UV based disinfection or therapeutic irradiation. UVC is increasingly used in clinical and commercial disinfection protocols due to its germicidal efficacy.^10^ However, the risks of exposure to human tissue, especially in medical settings or devices with inadequate shielding, should be reconsidered. If UVC radiation penetrates the tissues sufficiently to reach mitochondria, it may compromise bioenergetic function and promote undesirable cytotoxicity. However, UVC radiation does not have significant skin penetrability, but it should be considered that the eyes can be damaged by UVC. Interestingly, while most studies on phototherapy focus on DNA or reactive oxygen species related damage, there may be potentially parallel or even primary mitochondrial mechanism in phototoxicity. In the context of phototherapy, such as psoriasis or vitiligo treatment, where UVA or UVB is employed under controlled doses, the results suggest a relatively safe bioenergetic profile for UVA exposure supporting its continued clinical use with minimal mitochondrial risk.^11^

**Mitochondrial DNA and skin carcinogenesis:** Given that the skin is the primary tissue directly exposed to ultraviolet radiation, it is crucial to consider the specific detrimental changes induced in cutaneous mitochondria. This oxidative assault directly causes mutations in the circular mitochondrial DNA, which is highly vulnerable due to its lack of protective histones and limited repair mechanisms.^1^ The subsequent collapse in ATP production impairs critical cellular repair processes, while the amplified oxidative stress drives nuclear DNA damage and promotes pro-inflammatory signaling. This mitochondrial dysfunction is a fundamental driver in the pathogenesis of skin cancer. In melanoma, the most lethal form of skin cancer,^11^ oncogenic mutations often force cells to rely on mitochondrial respiration for energy, a process known as metabolic reprogramming. Conversely, in non-melanoma skin cancers, such as basal and squamous cell carcinoma, which account for over 5.4 million cases annually globally,^12^ UV-induced mitochondrial damage promotes a shift towards glycolytic metabolism even in the presence of oxygen, fueling rapid tumor proliferation. Therefore, the increasingly serious ultraviolet radiation is not just a surface-level threat; it is a profound environmental stressor that disrupts core mitochondrial bioenergetics, directly linking solar exposure to cellular metabolic dysfunction, genomic instability, and the escalating global burden of skin cancer.

**Mechanistic considerations and open questions:** The exact molecular targets of UVC within mitochondria remain to be elucidated. Is the damage caused by direct photon absorption by membrane proteins, such as components of Complex I–IV or ATP synthase? Does lipid peroxidation despite no statistical changes in UVA-exposed samples play a role at lower UVC thresholds? Are certain mitochondrial subpopulations more sensitive, depending on organ source or metabolic state? Moreover, while the use of isolated mitochondria provides clear mechanistic insights, *in vivo* systems incorporate layers of protection and repair (e.g., antioxidant enzymes, mitophagy, and tissue shielding). Thus, whether similar bioenergetic impairment occurs in intact cells, tissues, or organisms under clinically relevant UVC doses remains an open question. Additionally, the role of mitochondrial dysfunction in the mechanism of microbial photoinactivation deserves renewed attention. If mitochondria or their bacterial analogs are primary targets of UVC, this insight may refine the design of sterilization strategies and help differentiate lethal versus sublethal exposures.

**Conclusion:** The mitochondrion is more than just a passive target of oxidative stress functions as an active sensor and responder to photonic energy. With UV-based technologies becoming increasingly prevalent, from hospital disinfection systems to wearable phototherapy devices, understanding the full spectrum of biological responses at the organelle level is essential. This work establishes a foundation for reevaluating UV safety thresholds and therapeutic applications, emphasizing mitochondrial health as a key consideration.

*This work was supported by the National Council for Scientific and Technological Development (CNPq Proc. 130160/2021-0 to ASP, Brazilian Federal Agency for Support and Evaluation of Graduate Education (CAPES) to NMI and The Sao Paulo Research Foundation (FAPESP, CEPOF Proc. 2013/07276-1) (to VSB).*

## References

[1] Sharma N, Pasala MS, Prakash A (2019). Mitochondrial DNA: epigenetics and environment. Environ Mol Mutagen.

[2] Bottje WG (2019). Oxidative metabolism and efficiency: the delicate balancing act of mitochondria. Poult Sci.

[3] Schauen M, Hornig-Do HT, Schomberg S, Herrmann G, Wiesner RJ (2007). Mitochondrial electron transport chain activity is not involved in ultraviolet A (UVA)-induced cell death. Free Radic Biol Med.

[4] Barbatti M, Ullrich S, Borin AC (2015). Photoinduced phenomena in nucleic acids II DNA fragments and phenomenological aspects. Top Curr Chem.

[5] Birch-Machin MA, Swalwell H (2010). How mitochondria record the effects of UV exposure and oxidative stress using human skin as a model tissue. Mutagenesis.

[6] Perez AS, Oliveira CLP (2017). Thermal-induced denaturation and aggregation behavior of lysozyme and bovine serum albumin: a thermodynamic and structural study. Brazilian J Phys.

[7] Perez AS, Inada NM, Mezzacappo NF, Vollet-Filho JD, Bagnato VS (2024). Microwave radiation and thermal effects on the bioenergetics of isolated mitochondria. Int J Radiat Biol.

[8] Perez AS, Inada NM, Mezzacappo NF, Jose D (2024). VF, Bagnato VS. Ultraviolet radiation inhibits mitochondrial bioenergetics activity. Photochem Photobiol.

[9] Ronchi JA, Figueira TR, Ravagnani FG, Oliveira HCF, Vercesi AE, Castilho RF (2013). A spontaneous mutation in the nicotinamide nucleotide transhydrogenase gene of C57BL/6J mice results in mitochondrial redox abnormalities. Free Radic Biol Med.

[10] Gurzadyan GG, Gorner H, Schulte-Frohlinde D (1995). Ultraviolet (193, 216 and 254 nm) photoinactivation of Escherichia coil strains with different repair deficiencies. Radiat Res.

[11] Nakashima Y, Ohta S, Wolf AM (2017). Blue light-induced oxidative stress in live skin. Free Radic Biol Med.

[12] Hogue L, Harvey VM (2019). Basal cell carcinoma, squamous cell carcinoma, and cutaneous melanoma in skin of color patients. Dermatol Clin.

